# Osteoporosis After Menopause and After Drug Therapy: The Molecular Mechanism of Bone Loss and Its Treatment

**DOI:** 10.3390/ijms27020641

**Published:** 2026-01-08

**Authors:** Kelly I-Rong Lee, Jie-Hong Chen, Kuo-Hu Chen

**Affiliations:** 1 School of Medicine, College of Medicine, MacKay Medical University, New Taipei City 25245, Taiwan; kellya05.kl@gmail.com (K.I.-R.L.); albertjhc@gmail.com (J.-H.C.); 2 Department of Obstetrics and Gynecology, Taipei Tzu-Chi Hospital, The Buddhist Tzu-Chi Medical Foundation, New Taipei City 23142, Taiwan; 3 School of Medicine, Tzu-Chi University, Hualien 97004, Taiwan

**Keywords:** osteoporosis, postmenopausal osteoporosis, drug induced osteoporosis, treatment of osteoporosis

## Abstract

Osteoporosis is a prevalent skeletal disorder characterized by reduced bone mass and microarchitectural deterioration, leading to increased fracture risk, particularly in aging populations. Postmenopausal osteoporosis (PMOP) remains the most common primary form and results from abrupt estrogen deficiency after menopause, which disrupts bone remodeling by accelerating the receptor activator of nuclear factor-κB ligand (RANKL)-mediated osteoclastogenesis, suppressing Wnt/β-catenin signaling, and promoting inflammatory cytokine production. In contrast, drug-induced osteoporosis (DIOP) encompasses a heterogeneous group of secondary bone disorders arising from pharmacologic exposures. Glucocorticoids suppress osteoblastogenesis, enhance osteoclast activity, and increase reactive oxygen species; long-term bisphosphonate therapy may oversuppress bone turnover, resulting in microdamage accumulation; denosumab withdrawal triggers a unique rebound surge in RANKL activity, often leading to rapid bone loss and multiple vertebral fractures. Medications including aromatase inhibitors, SSRIs, proton pump inhibitors, heparin, and antiepileptic drugs impair bone quality through diverse mechanisms. Standard antiresorptive agents remain first-line therapies, while anabolic agents such as teriparatide, abaloparatide, and romosozumab provide enhanced benefits in high-risk or drug-suppressed bone states. Transitional bisphosphonate therapy is essential when discontinuing denosumab, and individualized treatment plans—including drug holidays, lifestyle interventions, and monitoring vulnerable patients—are critical for optimizing outcomes. Emerging approaches such as small interfering RNA (siRNA)-based therapeutics, anti-sclerostin agents, digital monitoring technologies, and regenerative strategies show promise for future precision medicine management. Understanding the distinct and overlapping molecular mechanisms of osteoporosis is essential for improving fracture prevention and long-term skeletal health.

## 1. Introduction

Osteoporosis is a systemic skeletal disorder characterized by reduced bone mass and microarchitectural deterioration, ultimately leading to increased bone fragility and fracture susceptibility [1]. Affecting more than 200 million individuals worldwide, it represents a major global health burden, particularly in aging populations [2]. As life expectancy continues to rise, the incidence of osteoporotic fractures—most commonly involving the hip, vertebrae, and wrist—is expected to increase substantially. These fractures contribute to significant morbidity, loss of independence, and excess mortality among older adults [3]. Although osteoporosis affects both sexes, postmenopausal women constitute a disproportionately high-risk group because the abrupt decline in estrogen at menopause accelerates bone turnover and favors bone resorption [4]. According to the International Osteoporosis Foundation, one in three women and one in five men over age 50 will sustain at least one osteoporotic fracture in their lifetime [5].

Postmenopausal osteoporosis (PMOP), the most common form of primary osteoporosis, is primarily driven by estrogen deprivation after menopause [1,6]. Estrogen is essential for skeletal homeostasis through its regulation of osteoclastogenesis, promotion of osteoblast survival, and maintenance of balanced bone remodeling via the receptor activator of nuclear factor-κB ligand (RANKL)/osteoprotegerin (OPG) system and the Wnt/β-catenin pathway [1,4,7,8]. Estrogen deficiency increases RANKL production while reducing OPG expression, thereby enhancing osteoclast differentiation and activity. In addition, estrogen withdrawal stimulates production of pro-inflammatory cytokines—including IL-1, IL-6, and TNF-α—further accelerating high-turnover bone loss [4,9,10].

In contrast, secondary osteoporosis, especially pharmacologically induced osteoporosis (DIOP), has become increasingly prevalent in modern clinical practice [11]. Long-term glucocorticoid exposure is one of the most common causes and leads to profound suppression of bone formation, increased osteoblast and osteocyte apoptosis, and inhibition of Wnt signaling [4,12]. More recently, denosumab withdrawal has emerged as a critical therapeutic challenge; its sudden cessation can cause rapid “rebound” activation of osteoclastogenesis through unopposed RANKL signaling, resulting in severe bone loss and clusters of vertebral fractures [13,14]. Additional drug classes—including aromatase inhibitors, proton pump inhibitors, selective serotonin reuptake inhibitors (SSRIs), heparin, and antiepileptic agents—have also been implicated in skeletal deterioration through diverse molecular mechanisms [15].

Although PMOP and DIOP share the final common pathway of skeletal fragility, they differ substantially in etiology, molecular drivers, clinical trajectory, and therapeutic considerations. PMOP typically follows a gradual, high-turnover bone loss pattern, whereas DIOP may manifest as either a low-turnover state (e.g., glucocorticoids, bisphosphonate oversuppression) or abrupt high-turnover crises (e.g., denosumab rebound) [4]. A major clinical challenge arises when these mechanisms coexist or when patients transition between different drug-induced states, for which current guidelines provide limited practical direction.

Recent advances in osteoporosis therapeutics have expanded treatment options beyond conventional antiresorptive agents. Romosozumab, a monoclonal antibody targeting sclerostin, and abaloparatide, a PTHrP analog, exert dual anabolic and antiresorptive effects, offering substantial improvements in bone mass and microarchitecture [16,17,18]. Furthermore, emerging non-pharmacological and technological approaches—including whole-body vibration therapy, wearable sensor–guided fall prevention, and nucleic acid-based therapeutics such as small interfering RNA (siRNA)—represent promising adjunctive strategies for enhancing bone regeneration and reducing fracture risk [19,20].

This review provides a comprehensive and integrated analysis of the molecular mechanisms, clinical manifestations, and therapeutic strategies underlying osteoporosis in two major settings: postmenopausal estrogen deficiency and drug-induced bone loss. By contrasting their pathophysiological foundations and treatment implications, we aim to clarify how personalized and mechanism-based interventions may optimize outcomes and advance precision medicine in osteoporosis care.

## 2. Methods of Literature Review

As a narrative review, the literature was searched to collect basic and clinical studies which investigated the molecular and cellular mechanism of osteoporosis after menopause and after drug therapy. For this review discussing the etiology of bone loss as well as its treatment, all of the articles had been solicited from the databases Ovid Medline and PubMed using the following search terms “osteoporosis”, “postmenopausal osteoporosis”, “drug induced osteoporosis” and “treatment of osteoporosis”. For screening and inclusion in the next stage, only English language articles with full text were considered for inclusion in a subsequent analysis. Duplicated articles were also excluded in the stage.

In the second stage, two experts in the field then inspected these articles to exclude studies with poor research design, questionable methods or unclear outcomes to ensure the quality of retrieved studies. Finally, a total of 174 articles were eligible for inclusion in this review.

## 3. Clinical Subtypes of Osteoporosis

### 3.1. Postmenopausal Osteoporosis (PMOP)

**Figure 1:**
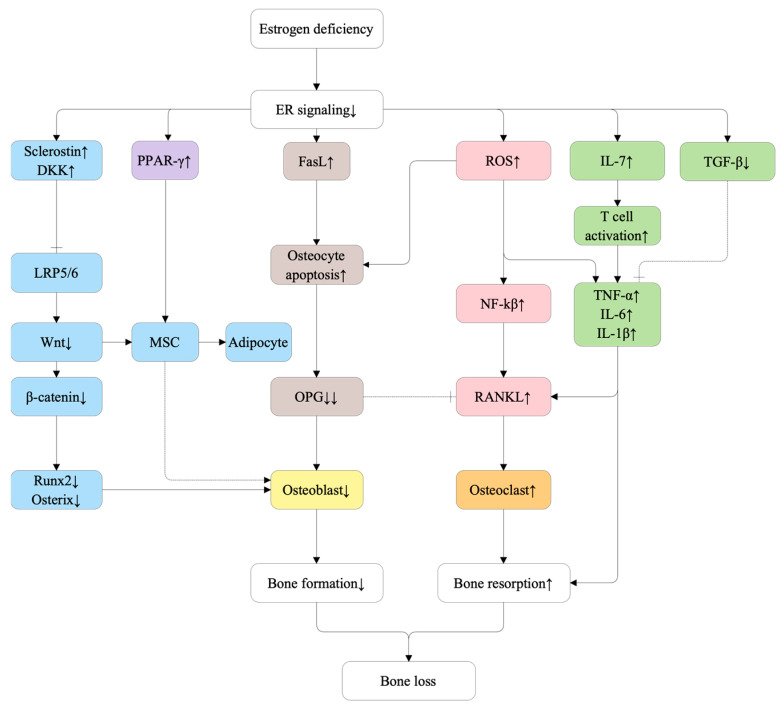
Molecular pathways involved in postmenopausal osteoporosis (PMOP). Estrogen deficiency reduces ERα signaling, increasing Fas ligand (FasL)–mediated osteocyte apoptosis and secretion of Wnt antagonists sclerostin and dickkopf-1 (DKK1), which inhibit Wnt/β-catenin signaling and downregulate RUNX2 and Osterix, impairing osteoblastogenesis. Reduced ERα signaling also increases peroxisome proliferator-activated receptor gamma (PPAR-γ) activity, promoting mesenchymal stem cell (MSC) differentiation toward adipocytes. In parallel, decreased transforming growth factor-beta (TGF-β) and increased interleukin-7 (IL-7) activate T cells, elevating pro-inflammatory cytokines (TNF-α, IL-1β, IL-6) that stimulate RANKL production. Estrogen deficiency elevates reactive oxygen species (ROS), which activate NF-κB to enhance RANKL expression, further enhancing osteoclast differentiation. The combined effects of increased RANKL/OPG ratio, reduced osteoblast number, and enhanced osteoclastogenesis lead to uncoupled bone remodeling and net bone loss. Solid arrows indicate direct stimulatory or inhibitory effects. Dashed inhibitory lines denote loss of inhibitory control mediated by decoy receptors (e.g., OPG), rather than direct suppression of ligand expression. ↑ and ↓ indicate relative increases or decreases compared with physiological baseline.

The molecular pathways involved in postmenopausal osteoporosis (PMOP) are illustrated in Figure 1.

Postmenopausal osteoporosis (PMOP) is the most common form of primary osteoporosis and predominantly affects women after menopause as a result of a marked decline in circulating estrogen levels [21]. Estrogen is essential for preserving skeletal integrity through its regulation of bone remodeling, modulation of osteoblast and osteoclast activity, and suppression of pro-inflammatory signaling [22]. Following menopause, the abrupt withdrawal of estrogen shifts the remodeling balance toward accelerated bone resorption, leading to a progressive decline in bone mineral density (BMD) [23].

The onset of PMOP is typically insidious, with cumulative bone loss occurring silently over many years before fragility fractures become clinically apparent [24]. Well-established risk factors include advancing age, early menopause (particularly before age 45), low body mass index (BMI), physical inactivity, insufficient intake of calcium or vitamin D, and a family history of osteoporosis [25]. A comprehensive summary of osteoporosis and fracture risk factors is presented in Table 1 [25,26,27,28,29,30]. Clinically, PMOP most commonly manifests as vertebral compression fractures, progressive loss of height, thoracic kyphosis, or low-energy fractures of the wrist or hip, particularly in elderly women [31,32]. Early identification of at-risk individuals through detailed clinical evaluation and BMD assessment is critical for timely intervention and fracture prevention [33].

### 3.2. Drug-Induced Osteoporosis (DIOP)

Drug-induced osteoporosis (DIOP) is a subtype of secondary osteoporosis that arises from prolonged exposure to specific pharmacologic agents or from the abrupt discontinuation of antiresorptive therapies [34]. In contrast to postmenopausal osteoporosis (PMOP), DIOP may develop more rapidly and often remains clinically silent until a fracture occurs, making early detection particularly challenging [34,35].

Glucocorticoids are the most extensively documented contributors to DIOP; they inhibit osteoblast differentiation, induce apoptosis of osteoblasts and osteocytes, and impair intestinal calcium absorption [4]. Other medications associated with significant skeletal deterioration include aromatase inhibitors used in breast cancer therapy, gonadotropin-releasing hormone (GnRH) agonists, and several antiepileptic drugs [36,37]. Notably, discontinuation of denosumab has been associated with an acute and severe rebound in bone resorption, frequently resulting in multiple vertebral fractures within months of cessation [38]. Additional agents—such as selective serotonin reuptake inhibitors (SSRIs), proton pump inhibitors (PPIs), Thiazolidinediones (TZDs), opioid, heparin, and various chemotherapeutic drugs—have also been implicated in accelerating bone loss through diverse mechanisms [39]. Because patients are often unaware of medication-related skeletal risks, osteoporosis may remain undiagnosed until a low-trauma fracture occurs [34]. Unlike PMOP, DIOP affects both men and women and can arise across a broader age spectrum.

Given the heterogeneous pathophysiology underlying DIOP, management strategies must be tailored to the specific pharmacologic agent and its mechanism of skeletal toxicity. A thorough medication history, baseline and follow-up bone mineral density (BMD) assessments, and careful monitoring for rebound dynamics following drug discontinuation are essential for early risk identification and prevention [33,40]. Clinical decision-making should balance the therapeutic benefits of the primary medication against its potential skeletal harm, integrating prophylactic interventions—such as calcium and vitamin D supplementation or timely initiation of antiresorptive therapy—when appropriate [41].

## 4. Molecular Biology, Physiology, and Pathophysiology of Postmenopausal Osteoporosis

### 4.1. Estrogen Signaling in Bone Metabolism

Estrogen is a central regulator of skeletal homeostasis, exerting its effects primarily through estrogen receptors alpha (ERα) and beta (ERβ), which are expressed in osteoblasts, osteoclasts, and osteocytes [42]. Among these receptor subtypes, ERα is the dominant mediator of estrogen’s osteoprotective actions, as evidenced by the profound skeletal abnormalities observed in ERα-knockout models, whereas ERβ deficiency results in comparatively minimal skeletal alterations [42,43]. Of the major endogenous estrogens—estrone (E1), estradiol (E2), and estriol (E3)—estradiol (E2) exhibits the highest affinity for estrogen receptors and is therefore the most potent regulator of downstream signaling events. Upon binding to ERα, estrogen initiates genomic signaling that modulates the transcription of key genes, including those encoding RANKL and osteoprotegerin (OPG), thereby suppressing osteoclast differentiation and promoting bone formation [4].

In addition to these nuclear effects, membrane-associated estrogen receptors mediate rapid non-genomic responses through activation of intracellular signaling cascades such as PI3K–Akt, MAPK/ERK, and c-Src [44]. These pathways contribute to osteoblast survival, cytoskeletal remodeling, and anti-apoptotic responses, providing an additional layer of regulatory control over bone cell activity [45,46]. This coordinated genomic and non-genomic signaling framework enables estrogen to exert both immediate and long-term effects on skeletal metabolism.

Furthermore, estrogen enhances osteoanabolic pathways, including the Wnt/β-catenin and bone morphogenetic protein (BMP) signaling networks, by promoting their activation and suppressing their endogenous antagonists [47]. For example, estrogen downregulates sclerostin, an osteocyte-derived inhibitor of Wnt signaling, thereby stabilizing β-catenin and facilitating osteoblast differentiation and function [48]. Estrogen also interacts with the TGF-β signaling axis to support efficient coupling between bone resorption and formation during the remodeling cycle [49,50]. Collectively, the multifaceted actions of estrogen through ERα, ERβ, and their downstream canonical and non-canonical pathways underscore its indispensable role in maintaining bone mass, microarchitecture, and skeletal integrity.

### 4.2. Bone Remodeling: Normal Physiology and Menopause-Related Disruption

Bone remodeling is a dynamic and lifelong physiological process essential for maintaining skeletal integrity and mineral homeostasis [51]. It proceeds through five highly coordinated phases—activation (recruitment of osteoclast precursors), resorption (osteoclast-mediated bone degradation), reversal (transition mediated by mononuclear cells), formation (osteoblast-driven matrix deposition and mineralization), and termination—that together ensure balanced turnover under normal hormonal conditions, particularly under the regulatory influence of estrogen [52,53].

A central molecular regulator of bone remodeling is the receptor activator of nuclear factor κB (RANK) signaling pathway [54]. RANK, expressed on osteoclast precursors, is activated by its ligand RANKL and inhibited by osteoprotegerin (OPG), a soluble decoy receptor. By competitively binding RANKL, OPG prevents osteoclastogenesis and suppresses resorptive activity [22]. Estrogen maintains skeletal homeostasis by promoting OPG expression and suppressing RANKL, thereby restraining osteoclast differentiation and enhancing osteoclast apoptosis in part through the stimulation of TGF-β production [55]. In estrogen-deficient states, however, RANKL expression increases while OPG decreases, shifting the remodeling balance toward osteoclast activation [22]. Osteocytes further contribute to this process by producing RANKL, amplifying osteoclast recruitment and activity. Osteocytes also regulate bone formation through modulation of the Wnt/β-catenin pathway. By secreting sclerostin and dickkopf-1 (DKK1), both potent Wnt antagonists, osteocytes suppress β-catenin signaling and inhibit osteoblast differentiation [48]. Estrogen normally downregulates these inhibitors, thereby supporting Wnt-mediated osteoanabolic signaling; however, estrogen withdrawal removes this restraint, allowing sclerostin and DKK1 to rise and further suppress osteoblastogenesis [48].

Following menopause, the abrupt decline in estrogen accelerates bone remodeling and shifts it toward a high-turnover state dominated by osteoclast activity [56]. Consistent with the molecular mechanisms described above, disruption of the RANKL/OPG balance favors excessive osteoclastogenesis, resulting in heightened bone resorption [57]. In parallel, estrogen deficiency functionally suppresses Wnt/β-catenin signaling, leading to impaired osteoblast differentiation and reduced bone formation, a process further compounded by increased oxidative stress and osteoblast dysfunction [48,55,58]. The combined effects of enhanced resorption and attenuated formation ultimately produce characteristic structural deterioration, including trabecular thinning, loss of trabecular connectivity, and increased cortical porosity [59]. These microarchitectural changes substantially increase skeletal fragility and fracture susceptibility, particularly at trabecular-rich sites such as the vertebral bodies and the proximal femur [33].

### 4.3. Immune and Inflammatory Mechanisms

Estrogen deficiency has profound immunological consequences and contributes to the pathogenesis of postmenopausal osteoporosis through osteoimmunological mechanisms [60]. In the absence of estrogen, activated T cells and macrophages produce elevated levels of pro-inflammatory cytokines—including tumor necrosis factor-α (TNF-α), interleukin-1 (IL-1), and interleukin-6 (IL-6)—which collectively enhance osteoclastogenesis and prolong osteoclast survival [61,62]. Estrogen withdrawal is also associated with increased interleukin-7 (IL-7), a potent stimulator of T-cell proliferation that amplifies TNF-α secretion and further intensifies inflammatory signaling [22]. These cytokines upregulate RANKL expression in osteoblasts and osteocytes while simultaneously suppressing osteoprotegerin (OPG), thereby exacerbating the RANKL/OPG imbalance that drives osteoclast activation [63,64].

In parallel, estrogen deficiency induces a marked rise in oxidative stress within the bone microenvironment [65]. Accumulation of reactive oxygen species (ROS) impairs osteoblast differentiation, promotes apoptosis of osteoblasts and osteocytes, and enhances osteoclast activity through redox-sensitive transcription factors [62,66]. ROS additionally stimulates TNF-α production and activates the NF-κB pathway, which further increases RANKL expression and accelerates osteoclast differentiation [59]. Mitochondrial dysfunction contributes to sustained ROS generation and suppresses Wnt/β-catenin signaling, thereby impairing osteoblastogenesis and perpetuating bone loss [67]. The convergence of amplified inflammatory cytokine signaling, NF-κB activation, and oxidative stress establishes a strongly pro-resorptive milieu. This environment accelerates trabecular deterioration, increases cortical porosity, and ultimately contributes to the heightened fragility fracture risk characteristic of postmenopausal osteoporosis [62,68].

### 4.4. Genetic and Environmental Factors

Genetic susceptibility plays a significant role in the onset and severity of postmenopausal osteoporosis (PMOP) [69]. Numerous single-nucleotide polymorphisms (SNPs) have been linked to reduced bone mineral density (BMD) and increased fracture risk, spanning genes central to bone metabolism. These include classic osteoporosis-associated genes such as ESR1, VDR, and COL1A1 [26,27,28]; Wnt-related regulators such as CTNNB1, LRP5, DAAM2, and WNT16 [62,68]; and emerging GWAS-identified loci including VTI1A and EPDR1 [70,71,72]. Additional contributors include the monogenic mutation PLS3 [73], as well as regulatory and transcriptional factors such as RANKL, ESR2, CREB1, ERBB2, and ING3 [26,72,74,75]. Epigenetic mechanisms—including DNA methylation, histone modifications, and non-coding RNAs—further modulate osteoblastogenesis and osteoclastogenesis, although their specific roles in PMOP continue to be elucidated [76].

Environmental and behavioral factors interact with genetic predispositions to further influence disease risk. Early menopause, inadequate intake of calcium or vitamin D, physical inactivity, low body mass index (BMI), cigarette smoking, excessive alcohol consumption, and a personal or family history of fractures are all well-established contributors to bone loss [77,78,79]. These factors affect endocrine regulation, nutrient metabolism, and mechanical loading of bone, thereby compounding fracture susceptibility. The interplay between underlying genetic architecture and modifiable lifestyle-related influences highlights the multifactorial nature of PMOP and underscores the need for comprehensive risk assessment and individualized prevention strategies [77,78]. Given the complexity of these signaling pathways, the key molecular mechanisms driving postmenopausal osteoporosis are summarized in Table 2.

## 5. Molecular and Cellular Mechanisms of Drug-Induced Osteoporosis

### 5.1. Glucocorticoid-Induced Osteoporosis (GIOP)

Glucocorticoids (GCs) induce osteoporosis primarily through multifaceted disruption of the molecular pathways governing bone remodeling. A central mechanism involves the suppression of osteoblastogenesis through downregulation of the osteogenic transcription factor RUNX2 and inhibition of Wnt/β-catenin signaling [80,81]. GCs increase the expression of Wnt antagonists—including Dickkopf-1 (DKK1) and sclerostin—and upregulate intracellular inhibitors such as Axin2 and GSK3β, which collectively destabilize β-catenin by impairing LRP5/6-mediated Wnt ligand activation. These changes prevent β-catenin nuclear translocation and suppress the transcription of osteoblast-related genes, thereby attenuating osteoblast differentiation and function [81,82,83]. In addition, GCs promote adipogenic commitment of mesenchymal stem cells (MSCs) through upregulation of PPAR-γ2, further reducing the pool of osteoprogenitors available for bone formation [84].

**Figure 2:**
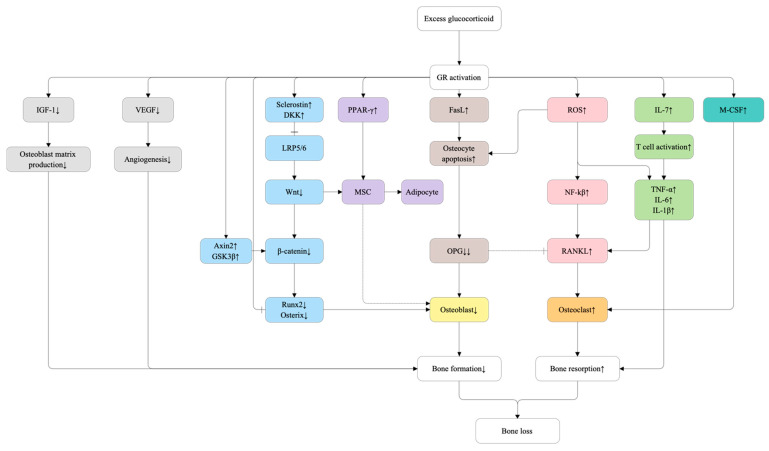
Molecular mechanisms of glucocorticoid-induced osteoporosis (GIOP). Excess glucocorticoid binds to the glucocorticoid receptor (GR), activating GR-dependent transcriptional regulation. GR activation directly upregulates sclerostin and Dickkopf-1 (DKK1), which inhibit the Wnt/β-catenin pathway by binding to LRP5/6, and increases AXIN2 and GSK3β expression, enhancing β-catenin degradation. Reduced β-catenin impairs transcription of osteogenic factors RUNX2 and Osterix, while GR also directly represses their transcription via glucocorticoid response elements (GREs), collectively suppressing mesenchymal stem cell (MSC) differentiation into osteoblasts. GR activation further increases PPAR-γ, favoring MSC adipogenic differentiation. In parallel, GR signaling elevates macrophage colony-stimulating factor (M-CSF), promoting osteoclastogenesis, and enhances reactive oxygen species (ROS) generation, which activates NF-κB and upregulates RANKL expression in osteoblast-lineage cells, further enhancing osteoclast differentiation. Elevated RANKL, together with markedly reduced osteoprotegerin (OPG), shifts the RANKL/OPG ratio toward osteoclast activation. Glucocorticoids also suppress vascular endothelial growth factor (VEGF), impairing angiogenesis, and reduce insulin-like growth factor-1 (IGF-1), limiting osteoblast matrix production. The convergence of Wnt/β-catenin suppression, osteoblast transcription factor inhibition, increased osteoclastogenic signaling, oxidative stress, and impaired bone vascular and matrix support results in decreased bone formation and increased bone resorption, culminating in net bone loss. Solid arrows indicate direct stimulatory or inhibitory effects. Dashed inhibitory lines denote loss of inhibitory control mediated by decoy receptors (e.g., OPG), rather than direct suppression of ligand expression.

Concurrently, GCs enhance osteoclastogenesis by increasing the expression of RANKL and macrophage colony-stimulating factor (M-CSF) while reducing osteoprotegerin (OPG) production, thus shifting the RANKL/OPG balance toward osteoclast differentiation and activation [84,85]. This dual disruption—suppression of anabolic Wnt signaling and amplification of catabolic RANKL signaling—creates a profoundly uncoupled remodeling environment characterized by excessive bone resorption alongside impaired bone formation [86]. Moreover, GCs downregulate vascular endothelial growth factor (VEGF), resulting in reduced skeletal angiogenesis and compromised bone strength [87]. The integrated molecular mechanisms underlying glucocorticoid-induced osteoporosis (GIOP) are summarized in Figure 2.

At the cellular level, glucocorticoids induce apoptosis in osteoblasts and osteocytes by activating pro-apoptotic members of the Bcl-2 family and suppressing key survival pathways such as PI3K/Akt [88,89]. This depletion of bone-forming cells disrupts the osteocytic network, which is essential for mechanosensation and coordination of bone turnover [90,91]. Glucocorticoids also impair mitochondrial function, leading to the accumulation of reactive oxygen species (ROS) [92]. Elevated ROS antagonizes β-catenin activity and activates NF-κB signaling in osteoclast precursors, thereby promoting osteoclastogenesis and amplifying bone resorption [65]. Moreover, glucocorticoids downregulate insulin-like growth factor-1 (IGF-1) signaling, an anabolic pathway critical for matrix synthesis, osteoblast differentiation, and cell survival [93].

At the epigenetic and transcriptional levels, glucocorticoids exert widespread gene-regulatory effects through glucocorticoid response elements (GREs), broadly repressing osteogenic transcriptional programs while enhancing catabolic signaling profiles [94]. This coordinated reprogramming further reinforces a skeletal microenvironment characterized by reduced bone formation and increased bone resorption [95].

### 5.2. Other Drug-Induced Mechanisms

A range of commonly used medications exert osteotoxic effects through diverse but mechanistically interconnected pathways.

#### 5.2.1. Proton Pump Inhibitors (PPIs)

Proton pump inhibitors (PPIs) may contribute to osteoporosis through several biological mechanisms. Reduced gastric acidity decreases the solubility and absorption of insoluble calcium salts, potentially impairing calcium homeostasis in susceptible individuals. Beyond effects on mineral absorption, PPIs directly inhibit osteoblast mineralization by suppressing PHOSPHO1, a phosphatase essential for matrix vesicle–mediated bone mineralization [96]. Recent genomic analyses further suggest that PPIs can perturb bone-cell signaling by altering the expression or activity of key regulatory genes—including EGFR, ESR1, and SRC—which are enriched in endocrine-resistance and ErbB signaling pathways, both of which intersect with osteogenic and survival signaling in bone cells [96,97,98].

**Figure 3:**
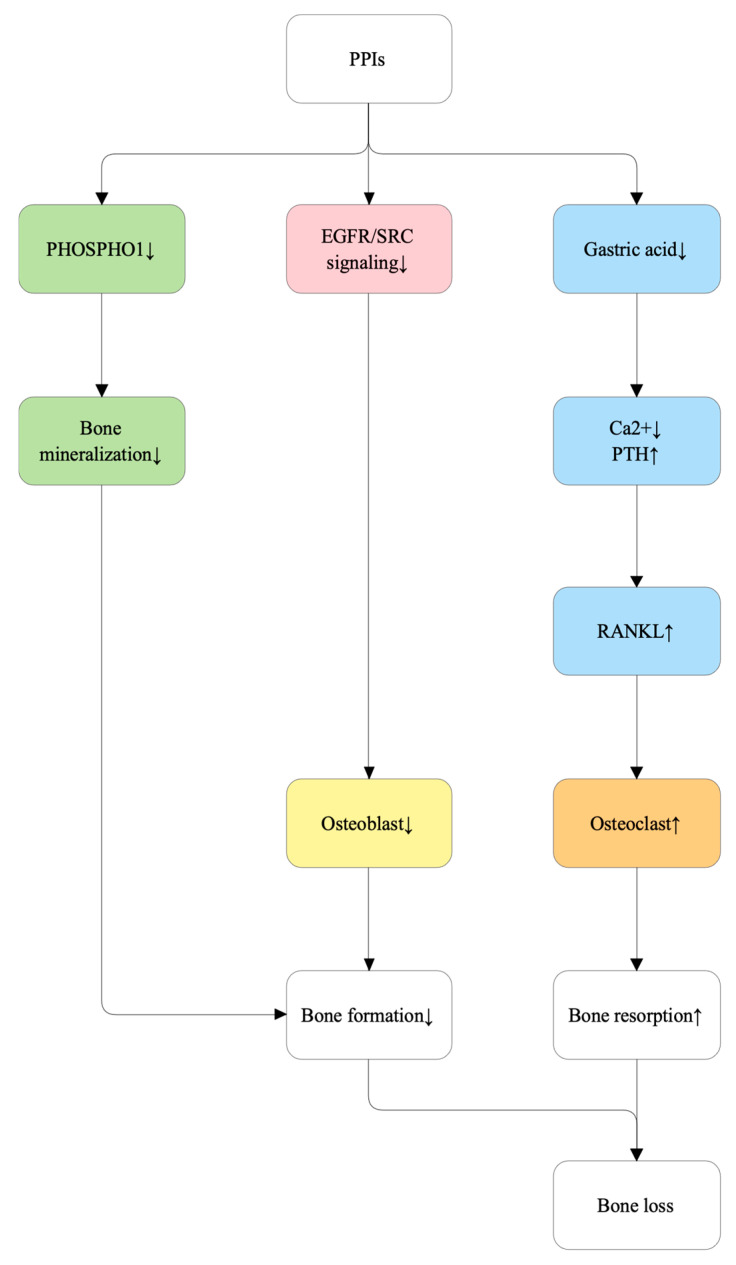
Proposed molecular mechanisms of proton pump inhibitor (PPI)-induced bone loss. Proton pump inhibitors (PPIs) contribute to skeletal fragility through multiple pathways. By suppressing gastric acid secretion, PPIs impair intestinal calcium absorption, leading to hypocalcemia and compensatory parathyroid hormone (PTH) elevation, which increases receptor activator of nuclear factor κB ligand (RANKL) expression and osteoclast-mediated bone resorption. In addition to indirect effects on calcium absorption, proton pump inhibitors directly impair osteoblast-mediated bone mineralization by suppressing PHOSPHO1, a key phosphatase required for matrix vesicle–dependent hydroxyapatite formation. PPIs also downregulate epidermal growth factor receptor (EGFR)/SRC signaling in osteoblasts, reducing osteoblast differentiation and bone formation. The combined effects of increased bone resorption and decreased bone formation culminate in net bone loss.

Secondary systemic effects such as hypergastrinemia, hypomagnesemia, and impaired vitamin B12 absorption may further disrupt bone remodeling and contribute to increased fracture risk, particularly with long-term therapy [97]. Figure 3 summarizes the proposed molecular mechanisms of PPI-induced bone loss.

#### 5.2.2. Antiepileptic Drugs (AEDs)

**Figure 4:**
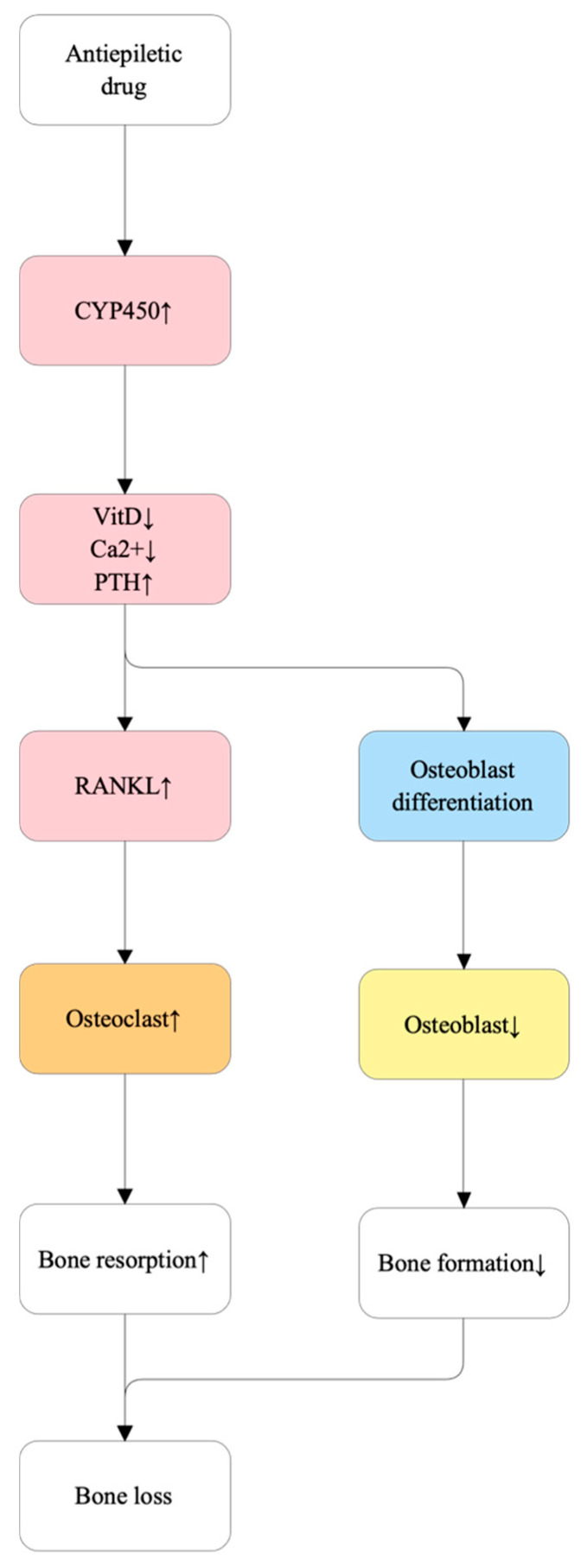
Molecular mechanisms of antiepileptic drug (AED)–induced bone loss. Enzyme-inducing AEDs accelerate cytochrome P450 (CYP450)-mediated vitamin D catabolism, leading to reduced serum vitamin D and calcium levels. This hypocalcemia triggers secondary hyperparathyroidism, elevating parathyroid hormone (PTH) levels and upregulating receptor activator of nuclear factor κB ligand (RANKL) expression, thereby promoting osteoclastogenesis and bone resorption. Concurrently, decreased vitamin D availability impairs osteoblast differentiation and reduces bone formation. The combined increase in bone resorption and decrease in bone formation disrupt bone remodeling, ultimately resulting in net bone loss.

Antiepileptic drugs (AEDs) accelerate cytochrome P450-mediated vitamin D catabolism, leading to hypocalcemia and a compensatory rise in parathyroid hormone (PTH) levels, which subsequently enhances osteoclast activity and bone resorption [99,100]. In addition to these endocrine disturbances, several AEDs directly impair osteoblast differentiation and bone formation by downregulating the synthesis of type I procollagen (COL1A1), a critical component of the bone matrix [101]. Furthermore, chronic AED therapy has been associated with hyperhomocysteinemia, which may independently alter bone microarchitecture and increase skeletal fragility [101]. Collectively, these metabolic and direct cellular effects disrupt the remodeling cycle and significantly elevate fracture risk [101]. The molecular mechanisms underlying AED-induced bone loss are summarized in Figure 4.

#### 5.2.3. SSRIs Modulate the Serotonin Transporter (SERT)

**Figure 5:**
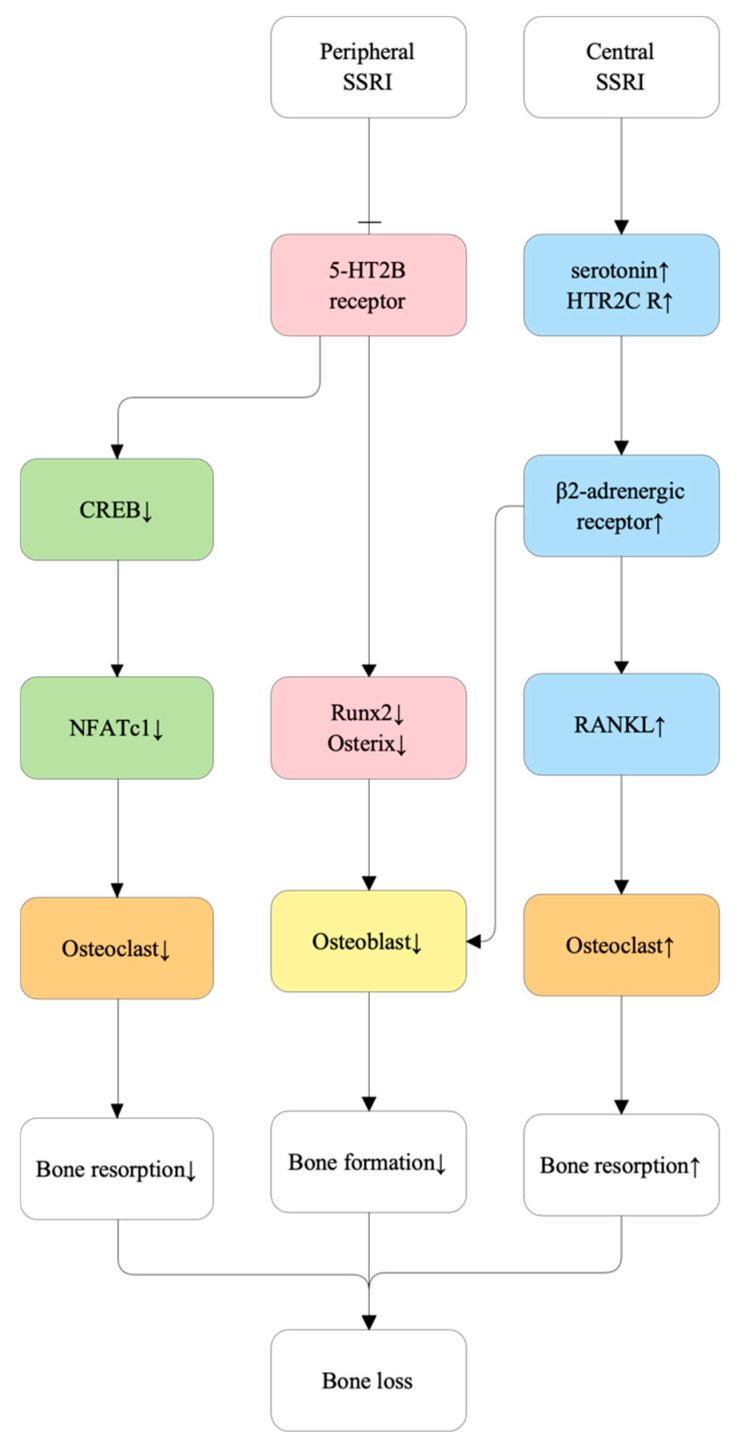
Molecular mechanisms under which selective serotonin reuptake inhibitors (SSRIs) affect bone metabolism. SSRIs influence bone remodeling through distinct peripheral and central pathways. Peripheral effects involve inhibition of the serotonin 5-HT2B receptor on osteoblasts and osteoclasts, reducing cAMP response element-binding protein (CREB) activation and subsequent nuclear factor of activated T-cells 1 (NFATc1) signaling, which suppresses osteoclastogenesis and lowers bone resorption. However, peripheral SSRI exposure downregulates the osteogenic transcription factors runt-related transcription factor 2 (Runx2) and Osterix, impairing osteoblast differentiation and reducing bone formation. Central effects are mediated via increased central serotonin levels and 5-HTR2C receptor activation, leading to desensitization-driven increases in sympathetic nervous system tone and β2-adrenergic receptor activation on bone cells. This enhances receptor activator of nuclear factor-κB ligand (RANKL) expression, stimulating osteoclastogenesis and elevating bone resorption, while also contributing to osteoblast suppression. The net effect of combined pathways is an imbalance between bone resorption and formation, ultimately leading to bone loss.

Selective serotonin reuptake inhibitors (SSRIs) alter bone metabolism through both serotonin transporter (SERT)–dependent and SERT-independent mechanisms. Beyond inhibiting SERT in osteoblasts and osteoclasts, SSRIs suppress the Ca^2+^/calmodulin–CREB–NFATc1 signaling cascade and perturb cAMP/PKA pathways, collectively impairing osteoblastogenesis, promoting apoptosis, and disrupting osteoclast function [102,103,104,105]. These alterations shift bone remodeling toward a net catabolic state, predisposing patients to bone loss and fragility fractures [103]. Figure 5 illustrates the proposed molecular pathways through which SSRIs influence bone remodeling.

#### 5.2.4. Thiazolidinediones (TZDs)

Thiazolidinediones (TZDs) exert adverse skeletal effects primarily through activation of the nuclear transcription factor peroxisome proliferator–activated receptor-γ (PPARγ), resulting in dysregulated bone remodeling [106]. Activation of PPARγ in bone marrow mesenchymal stem cells (MSCs) shifts lineage commitment toward adipogenesis at the expense of osteoblastogenesis, leading to reduced bone formation and increased accumulation of marrow adipose tissue [107]. In addition to suppressing osteoblast differentiation, TZDs promote bone resorption by enhancing osteoclast differentiation and activity, a process mediated in part by upregulation of receptor activator of nuclear factor-κB ligand (RANKL) and the osteoclastogenic transcription factor c-Fos [106,107]. Notably, experimental studies suggest that this interaction involves competition between PPARγ and estrogen receptor for the shared transcriptional coactivator steroid receptor coactivator-2 (SRC-2), thereby modulating osteoblast gene transcription and amplifying TZD-induced bone loss under estrogen-deficient conditions [106].

**Figure 6:**
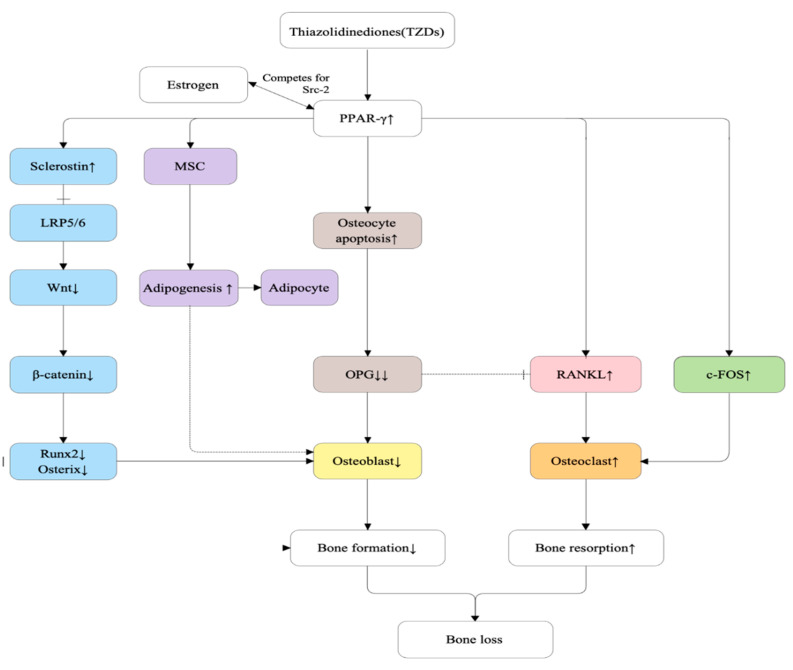
Molecular mechanisms underlying thiazolidinedione (TZD)–induced bone loss. TZDs activate peroxisome proliferator–activated receptor-γ (PPARγ), a nuclear receptor that disrupts skeletal homeostasis through coordinated effects on osteocytes, mesenchymal stem cells, and osteoclasts. Estrogen deficiency amplifies these effects by reducing estrogen receptor competition for the shared coactivator SRC-2, thereby enhancing PPARγ transcriptional activity. PPARγ promotes osteocyte apoptosis and increases sclerostin expression, suppressing Wnt signaling and bone formation; shifts mesenchymal stem cell differentiation toward adipogenesis at the expense of osteoblastogenesis; and upregulates c-Fos and RANKL, leading to increased osteoclast differentiation and bone resorption. Collectively, these mechanisms result in reduced bone formation, increased bone resorption, and an elevated risk of osteoporosis and fractures. Solid arrows indicate activation or upregulation. Dashed inhibitory lines denote loss of inhibitory control mediated by decoy receptors (e.g., OPG), rather than direct suppression of ligand expression.

Emerging evidence further indicates that TZDs induce osteocyte apoptosis and increase osteocyte-derived sclerostin expression, a potent inhibitor of the Wnt/β-catenin signaling pathway, thereby further suppressing osteoblastic activity and amplifying skeletal fragility [107]. Collectively, these mechanisms converge to create a low-formation, high-resorption state that accelerates bone loss and increases fracture risk in patients receiving TZD therapy [106,107]. Figure 6 shows the proposed mechanism of TZD-related bone loss.

#### 5.2.5. Opioids

Chronic opioid use is increasingly recognized as a contributor to osteoporosis through combined endocrine and direct skeletal mechanisms [108,109,110]. Centrally, activation of μ-opioid receptors suppresses the hypothalamic–pituitary–gonadal axis, reducing gonadotropin secretion and leading to decreased circulating sex hormones [108]. This functional hypogonadism mimics estrogen deficiency, resulting in increased RANKL expression, reduced osteoprotegerin (OPG) production, and enhanced osteoclastogenesis [55,109,110]. In parallel, opioid-induced reductions in testosterone further impair androgen receptor–mediated osteoblast activity and bone formation, thereby exacerbating opioid-associated skeletal fragility [111].

**Figure 7:**
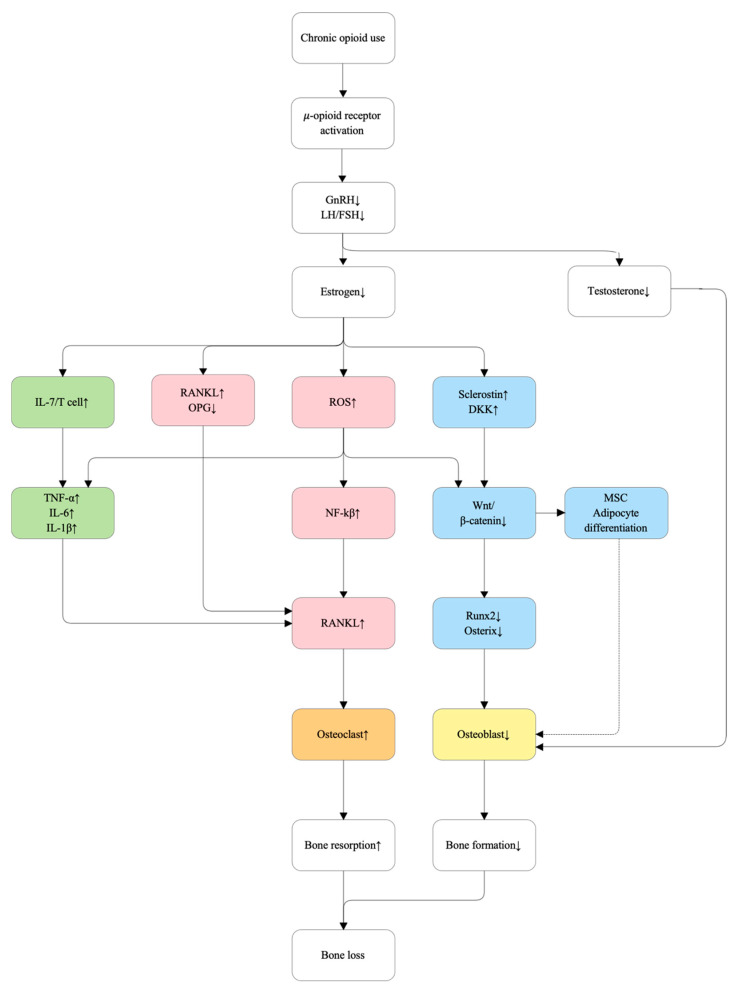
Proposed molecular mechanisms of opioid-induced bone loss. Chronic opioid exposure suppresses the hypothalamic–pituitary–gonadal axis, leading to reduced circulating sex hormones, particularly testosterone, thereby mimicking a hypogonadal state characterized by increased RANKL expression and decreased osteoprotegerin (OPG), which promotes osteoclastogenesis. In parallel, direct μ-opioid receptor signaling in osteocytes induces apoptosis and increases sclerostin expression, resulting in inhibition of Wnt/β-catenin signaling and suppression of osteoblast differentiation via downregulation of RUNX2 and Osterix. Opioid-induced oxidative stress further activates NF-κB signaling, amplifying RANKL-mediated osteoclast differentiation and bone resorption. Collectively, these endocrine and cell-intrinsic mechanisms uncouple bone remodeling and accelerate bone loss during long-term opioid therapy.

In addition to hormonal effects, opioids may exert direct actions on bone cells [110]. μ-Opioid receptors are expressed in osteocytes and osteoblasts, and experimental studies suggest that opioid exposure increases oxidative stress and osteocyte apoptosis [110,112]. These changes can impair Wnt/β-catenin signaling and suppress osteoblast differentiation through downregulation of RUNX2 and Osterix [48,62,110]. Opioid-induced oxidative stress further activates NF-κB signaling, thereby amplifying RANKL-mediated osteoclast differentiation and activity [62,112]. Collectively, these mechanisms uncouple bone remodeling by simultaneously suppressing bone formation and enhancing bone resorption, contributing to accelerated bone loss during long-term opioid therapy [52,62,108,110]. Figure 7 illustrates the proposed molecular mechanisms underlying opioid-associated bone loss.

#### 5.2.6. Aromatase Inhibitors (AIs)

**Figure 8:**
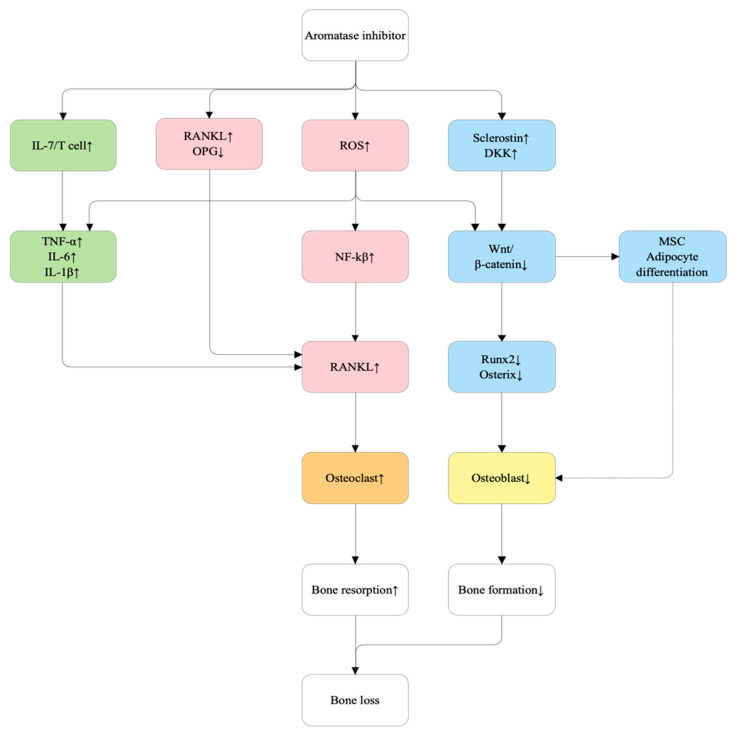
Proposed molecular mechanisms of aromatase inhibitor (AI)-induced bone loss. Aromatase inhibitors (AIs) suppress estrogen biosynthesis, resulting in estrogen deficiency and downstream disruption of bone remodeling. Reduced estrogen signaling increases interleukin-7 (IL-7) production and T-cell activation, elevating pro-inflammatory cytokines (TNF-α, IL-6, IL-1β) that enhance receptor activator of nuclear factor κB ligand (RANKL) expression and osteoclastogenesis. Estrogen deficiency also reduces osteoprotegerin (OPG) levels, shifting the RANKL/OPG ratio toward bone resorption. Concurrently, elevated reactive oxygen species (ROS) activate NF-κB to further increase RANKL expression. Estrogen deficiency upregulates Wnt antagonists sclerostin and Dickkopf-1 (DKK1), which inhibit Wnt/β-catenin signaling, leading to downregulation of osteogenic transcription factors RUNX2 and Osterix, impaired osteoblast differentiation, and increased mesenchymal stem cell (MSC) commitment toward adipocytes. The combined effects of increased osteoclast activity and reduced osteoblast-mediated bone formation lead to uncoupled remodeling and net bone loss.

Aromatase inhibitors (AIs) suppress estrogen synthesis, resulting in the loss of estrogen-mediated protection on bone. Reduced estrogen signaling leads to unchecked RANKL expression, increased production of IL-6, and downregulation of key osteoanabolic pathways such as Wnt/β-catenin and BMP signaling [47,57,61,62,113]. Collectively, these molecular disruptions contribute to AI-induced osteoporosis. Figure 8 shows the proposed mechanism of AI-related bone loss.

#### 5.2.7. Heparin

**Figure 9:**
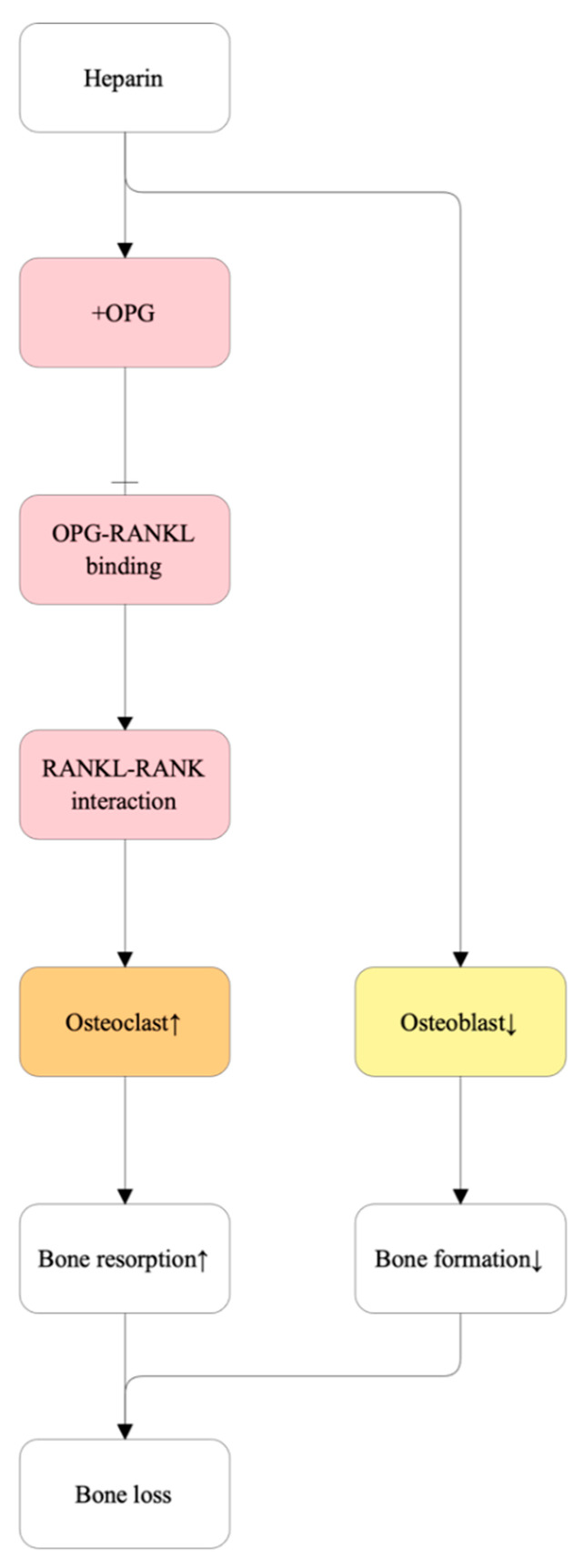
Proposed molecular mechanism of heparin-induced osteoporosis. Heparin binds to osteoprotegerin (OPG), reducing its ability to sequester receptor activator of nuclear factor κB ligand (RANKL). The resulting decrease in OPG–RANKL binding enhances RANK–RANKL interactions, thereby promoting osteoclast differentiation and activity, leading to increased bone resorption. Concurrently, heparin exerts inhibitory effects on osteoblast activity, further reducing bone formation. The combined effects of elevated bone resorption and suppressed bone formation result in net bone loss.

Heparin promotes bone resorption primarily by binding to osteoprotegerin (OPG), thereby preventing OPG from sequestering RANKL and resulting in unopposed RANK–RANKL signaling [114,115]. This shift in the RANKL/OPG balance enhances osteoclast differentiation and activity. Unfractionated heparin (UFH) exhibits more pronounced skeletal effects than low-molecular-weight heparin (LMWH), largely due to its higher binding affinity for OPG and greater degree of biological interaction within the bone microenvironment [114,116]. In addition to its effects on osteoclast regulation, heparin suppresses osteoblast activity and may perturb anabolic pathways involving TGF-β and BMP/Wnt signaling; however, the dominant outcome is a net increase in bone resorption that ultimately contributes to reduced bone mass and skeletal fragility [117]. Figure 9 illustrates the proposed molecular mechanisms underlying heparin-induced osteoporosis.

### 5.3. Denosumab Withdrawal and RANKL Rebound

**Figure 10:**
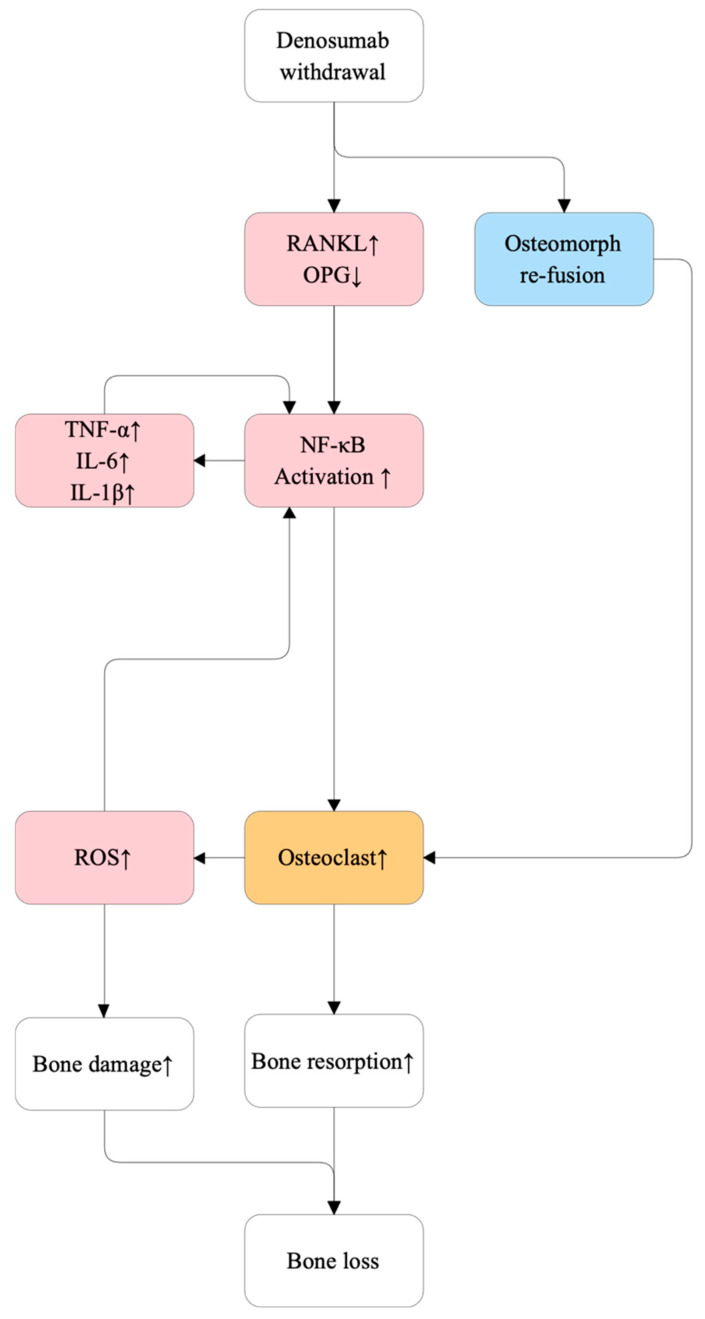
Molecular mechanisms of bone loss following denosumab withdrawal. Denosumab discontinuation rapidly reverses its antiresorptive effect, leading to a rebound increase in RANKL and a decrease in osteoprotegerin (OPG), thereby shifting the RANKL/OPG balance toward osteoclastogenesis. In parallel, osteomorphs—osteoclast-derived cells generated during therapy—re-fuse into mature osteoclasts, further amplifying osteoclast number and activity. Elevated IL-7 promotes T cell activation, which enhances the production of pro-inflammatory cytokines (TNF-α, IL-6, IL-1β) that synergize with RANKL signaling to stimulate osteoclast differentiation and survival. Activated osteoclasts increase bone resorption and generate reactive oxygen species (ROS), which both activate NF-κB–dependent pro-resorptive pathways and directly damage bone matrix. The combined effects of accelerated bone resorption and ROS-induced bone damage result in rapid bone loss and increased fracture risk.

Denosumab is a fully human monoclonal antibody with high specificity for RANKL, preventing its interaction with RANK on osteoclast precursors and thereby inhibiting osteoclast formation, function, and survival [118]. This results in profound suppression of bone resorption and substantial gains in bone mineral density (BMD) during treatment [119,120]. However, because denosumab does not bind to the bone matrix, its antiresorptive effects dissipate rapidly once therapy is discontinued. This leads to a rebound surge in available RANKL, reactivation of osteoclast precursors, and an abrupt acceleration of bone remodeling, often exceeding pre-treatment levels [119,121]. Recent findings show that osteomorphs—osteoclast-derived precursor-like cells that accumulate during denosumab therapy—can rapidly re-fuse into mature osteoclasts upon discontinuation, further amplifying bone resorption [122]. Clinically, this rebound phenomenon is associated with a markedly increased risk of multiple vertebral fractures, especially in trabecular-rich skeletal sites [121]. Figure 10 depicts the molecular mechanisms driving bone loss after denosumab withdrawal.

From a molecular standpoint, rebound bone loss following denosumab cessation may be further exacerbated by osteoimmune interactions [121]. Under conditions of estrogen deficiency or pharmacologic perturbation, activated T cells and B cells contribute to increased RANKL production, while pro-inflammatory cytokines—including IL-6 and TNF-α—synergize with RANKL signaling to enhance osteoclast differentiation and prolong osteoclast survival [121,123,124,125]. Concurrent reductions in OPG after treatment cessation further tilt the RANKL/OPG balance toward osteoclastogenesis [119,121]. This imbalance activates nuclear factor kappa-B (NF-κB), a master regulator of osteoclast differentiation and function [126]. Excessive osteoclast activity also elevates reactive oxygen species (ROS), which contribute directly to bone matrix damage and indirectly reinforce pro-resorptive pathways via further NF-κB activation [127]. Elevated pro-inflammatory cytokines such as TNF-α, IL-6, and IL-1β downstream of NF-κB signaling additionally promote osteoclastogenesis and bone resorption following denosumab withdrawal [126,128]. Although immune-mediated amplification is not the primary trigger of rebound bone loss, it may worsen outcomes in aging individuals with underlying inflammatory dysregulation [121,125]. Together, these mechanisms characterize denosumab withdrawal as a distinct, pharmacologically induced RANKL-driven osteolytic crisis, underscoring the need for transitional antiresorptive therapy—most commonly bisphosphonates—to prevent skeletal deterioration [119].

### 5.4. Bisphosphonate Oversuppression and Microdamage Accumulation

Bisphosphonates (BPs) inhibit osteoclast activity by targeting farnesyl pyrophosphate synthase (FPPS), disrupting cytoskeletal organization and inducing osteoclast apoptosis [129,130]. While initially protective, prolonged suppression of bone turnover impairs the skeleton’s ability to repair microdamage, especially within cortical bone [131]. Adequate bone turnover is required for removing aged, microdamaged bone; chronic oversuppression reduces both resorption and formation, lowering overall remodeling capacity [132]. Experimental models demonstrate that extended suppression leads to the accumulation of linear microcracks, compromising bone mechanical integrity and predisposing to structural failure [131,132].

**Figure 11:**
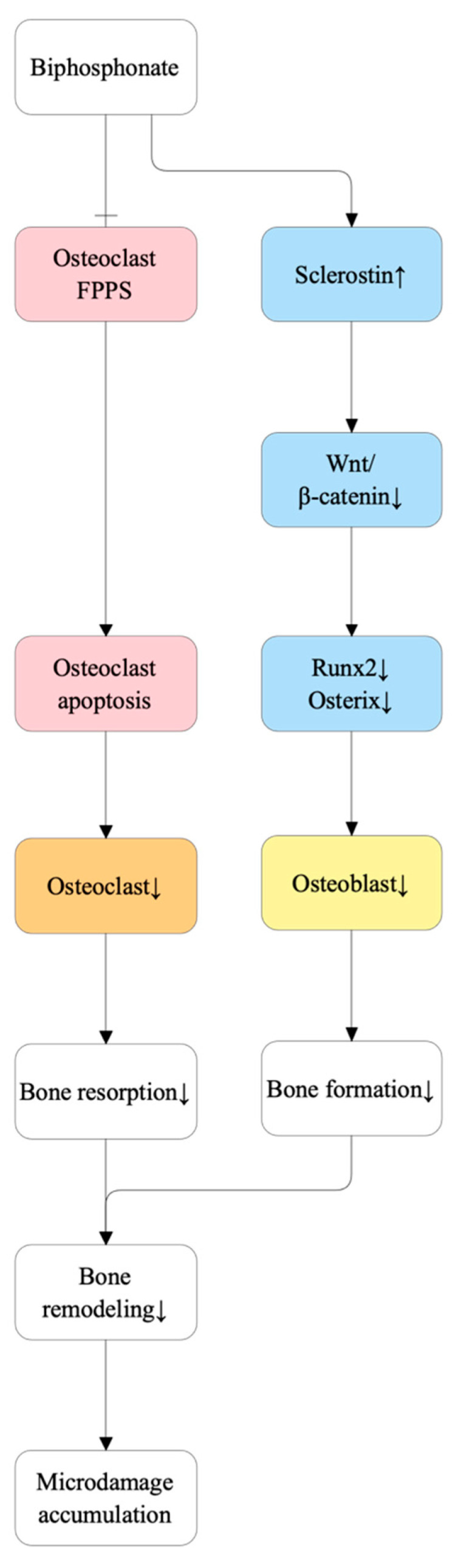
Proposed mechanism of bone loss induced by bisphosphonates. The diagram illustrates two primary pathways through which bisphosphonates may disrupt bone metabolism. The first pathway involves the inhibition of farnesyl pyrophosphate synthase (FPPS) within osteoclasts, leading to osteoclast apoptosis, reduced osteoclast numbers, and decreased bone resorption. The second pathway shows that bisphosphonates can increase the expression of Sclerostin, which suppresses the Wnt/β-catenin signaling pathway. This in turn downregulates osteogenic transcription factors Runx2 and Osterix, resulting in impaired osteoblast differentiation and reduced bone formation. Both decrease in bone resorption and formation can contribute to an overall decline in bone remodeling. This prolonged suppression of bone remodeling ultimately leads to the accumulation of microdamage within the bone matrix, potentially compromising skeletal integrity.

Prolonged remodeling suppression also alters osteocyte-mediated signaling. Suppressed turnover elevates sclerostin levels, inhibiting Wnt/β-catenin signaling and impairing osteoblast differentiation and matrix formation [133,134,135]. Downregulation of Wnt signaling subsequently reduces RUNX2 and Osterix expression, further impairing osteoblastogenesis [136]. This sustained low-turnover state promotes matrix aging and advanced collagen cross-linking, diminishing bone toughness and increasing susceptibility to atypical femoral fractures (AFFs), particularly in the subtrochanteric region with minimal trauma [115,137,138]. Importantly, prolonged suppression of bone remodeling has also been implicated in the pathogenesis of osteonecrosis of the jaw (ONJ), a rare but serious complication characterized by impaired bone healing following dental trauma or infection [33,129]. Low bone turnover, reduced osteoclast-mediated remodeling, and compromised microdamage repair are thought to impair jawbone resilience, particularly in the setting of local inflammatory or mechanical stress [139,140]. Additional contributing factors—including advanced age, long-term glucocorticoid co-treatment, diabetes, and alterations in collagen cross-linking—may further heighten skeletal fragility [141,142,143]. Current therapeutic guidelines therefore recommend periodic reassessment of bisphosphonate treatment duration and consideration of “drug holidays” in low-risk patients to restore physiological remodeling dynamics [144]. Figure 11 summarizes the proposed mechanism of bisphosphonate-induced bone loss.

Although individual drug mechanisms differ, these agents collectively destabilize the tightly regulated balance of bone remodeling. Cross-talk among metabolic, endocrine, and local signaling pathways further amplifies skeletal vulnerability, particularly in older adults or those exposed to polypharmacy. Given the wide range of pharmacological agents and the complexity of their underlying mechanisms, Table 3 provides a comprehensive summary of the specific molecular pathways and the associated impact on bone remodeling for all drug classes discussed in this section.

While elucidating the molecular mechanisms of drug-induced osteoporosis is fundamental, translating this knowledge into clinical practice requires identifying susceptible patient profiles and recognizing the thresholds for intervention. To this end, Table 4 summarizes the clinical risk factors, typical dosages, and treatment durations associated with clinically significant bone loss for the pharmacologic agents discussed [31,109,145,146,147,148]. This consolidation is particularly critical for widely prescribed medications such as proton pump inhibitors (PPIs) and antiepileptic drugs, where fracture risk is often underestimated in the general population compared to well-established offenders like glucocorticoids. To avoid redundancy with general osteoporosis risk factors (e.g., age, smoking, low BMI) discussed in previous section, this table highlights only those factors that specifically potentiate the adverse skeletal effects of the respective pharmacotherapies.

## 6. Treatment Strategies and Comparative Therapeutic Approaches

### 6.1. Standard Pharmacological Therapies

Antiresorptive therapies remain the foundation of osteoporosis management [26,80]. Bisphosphonates and denosumab, a monoclonal antibody targeting RANKL, effectively suppress osteoclast-mediated bone resorption, whereas selective estrogen receptor modulators (SERMs) provide particular benefit in postmenopausal osteoporosis (PMOP) owing to their estrogen-agonistic effects on bone [25,118,149]. In drug-induced osteoporosis (DIOP)—including glucocorticoid-induced osteoporosis (GIOP)—antiresorptives are similarly employed; however, management requires careful monitoring due to the potential for rapid bone turnover recovery following drug discontinuation, as exemplified by the rebound phenomenon associated with denosumab withdrawal [119,150].

Individualized therapy is essential and should consider patient age, renal function, fracture risk, comorbidities, and medication tolerance [33,129,151]. Long-term bisphosphonate treatment warrants periodic reassessment because of rare but serious adverse events, including atypical femoral fractures and osteonecrosis of the jaw (ONJ) [35]. When discontinuing denosumab, sequential bisphosphonate therapy is recommended to mitigate rebound bone loss and prevent vertebral fractures [119].

Anabolic agents—including teriparatide, abaloparatide, and romosozumab—directly stimulate bone formation and are particularly beneficial for patients at very high fracture risk or those with severe GIOP, in whom osteoblast function is profoundly suppressed [25,35,150]. These agents achieve more rapid and substantial gains in bone density than antiresorptives. For patients with severe osteoporosis, sequential regimens beginning with an anabolic agent followed by antiresorptive consolidation are recommended [33,120].

### 6.2. Emerging and Adjunctive Therapies

Emerging therapies aim to address limitations of standard pharmacologic options and broaden the scope of individualized care. Whole-body vibration therapy (WBVT) and virtual reality-based rehabilitation can improve neuromuscular performance, thereby enhancing bone strength and reducing fall risk [152,153]. Wearable sensor technologies enable continuous assessment of mobility and fall propensity, providing opportunities for personalized, data-driven interventions [154,155].

Nucleic acid-based therapeutics, particularly small interfering RNAs (siRNAs) targeting key regulators such as sclerostin or DKK1, represent a promising frontier by enabling selective modulation of osteogenic pathways and Wnt signaling [156,157]. This mechanism parallels the biologic action of romosozumab, an anti-sclerostin monoclonal antibody that simultaneously enhances bone formation and reduces resorption.

Bioactive compounds derived from traditional Chinese medicine—many with anti-inflammatory, antioxidant, and osteoanabolic properties—are increasingly evaluated in clinical and translational studies [158,159]. Gene therapy and stem-cell-based approaches have demonstrated potential in preclinical models but require further investigation before clinical translation [156].

### 6.3. Non-Pharmacological Interventions

Non-pharmacological strategies form an essential component of comprehensive care for both PMOP and DIOP [31,34]. Weight-bearing and resistance exercise enhances osteogenesis and improves muscle strength, contributing to fall prevention [25,33]. Adequate calcium, vitamin D, and protein intake supports bone mineralization and skeletal repair [25,41]. Fall prevention measures—such as home environment modifications, balance training, and hazard mitigation—are crucial in reducing fracture incidence [31,33].

Lifestyle modifications, including smoking cessation and limiting alcohol consumption, significantly improve bone health [25]. Appropriate sunlight exposure should be encouraged to support endogenous vitamin D synthesis while balancing skin cancer risk [41].

### 6.4. Treatment Matching and Drug

Optimal treatment selection requires alignment with the underlying pathophysiology of the disease [33,34]. In PMOP, which is driven by estrogen deficiency leading to increased RANKL signaling and reduced Wnt/β-catenin activity, hormone replacement therapy, bisphosphonates, or denosumab remain first-line interventions, followed by maintenance therapy to preserve bone mass [31,33,34,150].

In contrast, DIOP necessitates mechanism-specific treatment. In GIOP, early initiation of anabolic therapy (teriparatide, abaloparatide, or romosozumab) is recommended to counteract profound suppression of osteoblast function [34,35,39,150]. When feasible, discontinuing or reducing exposure to the offending drug is the most effective strategy for mitigating skeletal harm.

For patients discontinuing denosumab, sequential bisphosphonate therapy is essential to suppress rebound RANKL activation and prevent vertebral fractures [40]. Other DIOP entities require targeted interventions: PPI-associated bone loss warrants correction of calcium and vitamin D deficiency; AED-induced osteoporosis may require supplementation and antiresorptive therapy; and heparin-induced bone loss may be mitigated by transitioning from unfractionated heparin (UFH) to low-molecular-weight heparin (LMWH) [36,39,101].

Bisphosphonate drug holidays may be considered for low-risk patients after 3–5 years of therapy, whereas individuals with GIOP usually require continuous therapy until glucocorticoid tapering restores bone balance [33,35,150].

Economic constraints and treatment adherence—shaped by regimen complexity, medication costs, limited disease awareness, and concerns about side effects—substantially influence therapeutic sequencing and long-term success [31,33,150].

### 6.5. Influence of Comorbidities on Treatment Decisions

Beyond hormone- or drug-mediated mechanisms, osteoporosis should be viewed as a disease embedded within a broader multimorbidity context [160]. In clinical settings, osteoporosis rarely occurs in isolation; instead, it coexists with metabolic, cardiovascular, renal, and inflammatory disorders that independently affect skeletal integrity [161]. Failure to account for these comorbidities may lead to underestimation of fracture risk and suboptimal therapeutic decision-making, particularly in patients receiving long-term pharmacologic treatments [33].

Diabetes, chronic kidney disease, obesity, and cardiovascular disease alter bone quality, modify drug response, and increase fracture risk [162,163,164]. These conditions should direct clinicians toward earlier screening, preference for medications with safer metabolic profiles, and closer monitoring [33].

## 7. Discussion

Bone loss resulting from estrogen deficiency, glucocorticoid excess, denosumab withdrawal, and long-term pharmacotherapy with agents such as bisphosphonates, selective serotonin reuptake inhibitors (SSRIs), proton pump inhibitors (PPIs), heparin, or antiepileptic drugs shares several convergent mechanisms [22,88,97,103,116,119,143,144]. Central among these are dysregulation of the RANKL/OPG axis, suppression of Wnt/β-catenin signaling, and the accumulation of reactive oxygen species (ROS), all of which contribute to uncoupled bone remodeling [21,50,124,165]. These pathways highlight the inherent vulnerability of skeletal homeostasis to both endocrine alterations and pharmacological perturbations [34,50].

Although these mechanisms converge on similar downstream pathways, the initiating triggers and dominant molecular disruptions differ substantially across etiologies. Denosumab withdrawal is marked by abrupt reactivation of the RANKL–RANK pathway and osteomorph re-fusion—phenomena uniquely attributable to pharmacologic RANKL inhibition [119]. Bisphosphonate therapy, in contrast, induces sustained oversuppression of turnover, leading to microdamage accumulation rather than excessive osteoclastogenesis [149]. SSRIs exert both peripheral impairment of CREB/NFATc1-mediated osteoblast and osteoclast signaling and central enhancement of sympathetic β2-adrenergic tone, increasing RANKL expression [104]. PPIs and heparin primarily alter mineral homeostasis or extracellular protein interactions such as calcium solubility, phosphate balance, or OPG sequestration, while antiepileptic drugs accelerate vitamin D catabolism and impair osteoblast differentiation [34,101,117]. Together, these distinctions demonstrate that drug-induced osteoporosis cannot be conceptualized as a single mechanistic entity but instead reflects diverse and drug-specific perturbations [34,36].

Beyond hormone- or drug-mediated mechanisms, osteoporosis is fundamentally a multimorbidity-associated condition, a factor underemphasized in many prior reviews and clinical discussions. Several common chronic diseases—particularly type 2 diabetes mellitus (T2DM), hypertension, obesity, chronic kidney disease, and rheumatoid arthritis—independently alter bone quality, modify bone turnover, and significantly influence fracture risk [162,163,164,166].

Patients with T2DM exhibit paradoxically normal or elevated BMD yet sustain higher fracture rates due to impaired microarchitecture, accumulation of advanced glycation end-products (AGEs), reduced bone turnover, and altered osteocyte function [167]. Chronic kidney disease (CKD) is frequently accompanied by CKD–mineral and bone disorder (CKD-MBD), a systemic disturbance of calcium, phosphate, parathyroid hormone, and vitamin D metabolism that progressively impairs bone strength and markedly increases fracture risk as renal function declines [168]. Hypertension is associated with increased fracture risk through mechanisms involving chronic low-grade inflammation, vascular calcification, and disturbed calcium homeostasis, reflecting shared pathophysiological pathways between arterial calcification and skeletal demineralization [169]. Obesity, while traditionally considered protective, is increasingly recognized as a state of chronic low-grade inflammation and altered adipokine signaling that can exacerbate osteoblast suppression and dysregulate MSC differentiation [170,171]. Comorbidities and polypharmacy can compound fracture risk and complicate osteoporosis prevention and treatment decisions, beyond the effect of any single exposure [141,168,169,172,173]. Accordingly, osteoporosis should be approached as a comorbidity-driven disorder requiring comprehensive clinical evaluation and tailored therapeutic decision-making.

Medication-induced osteoporosis remains a particularly underrecognized contributor to fracture risk in clinical practice [31,101]. Long-term exposure to antiepileptic drugs (AEDs)—including phenytoin, carbamazepine, and valproate—has been consistently associated with reduced BMD and increased fracture rates [101]. AEDs induce CYP450-mediated vitamin D catabolism, leading to secondary hyperparathyroidism and enhanced osteoclast activity, while simultaneously impairing osteoblast differentiation and bone formation [101]. These findings indicate that AED-related bone loss is not solely metabolic but also reflects direct interference with osteogenic pathways [101,145]. Routine monitoring of vitamin D status and early implementation of supplementation should therefore be considered standard components of long-term AED therapy [101,145].

Other commonly prescribed medications also warrant vigilance. Proton pump inhibitors (PPIs), widely used for gastrointestinal disorders, can impair the absorption of insoluble calcium (e.g., calcium carbonate) by suppressing gastric acid secretion, thereby increasing the risk of hip and vertebral fractures with long-term use [174]. Furthermore, patients receiving long-term heparin therapy—such as those managing thromboembolic risks—may experience suppressed osteoblast formation and increased bone resorption [148]. Recognizing these iatrogenic risks allows physicians to consider alternative treatments or initiate early bone-protective strategies [31].

The mechanistic diversity underlying osteoporosis carries direct and clinically meaningful implications. For example, denosumab discontinuation requires transitional antiresorptive therapy—typically a bisphosphonate—to blunt rebound osteoclastogenesis and prevent vertebral fractures [119]. Conversely, long-term bisphosphonate therapy necessitates periodic reassessment and strategic drug holidays to mitigate the risk of atypical femoral fractures [33,149]. Patients treated with SSRIs, PPIs, heparin, or AEDs may require earlier and more frequent BMD monitoring, particularly when additional skeletal risk factors such as advanced age, menopause, glucocorticoid use, or endocrine comorbidities are present [35,96,101,103,116].

Importantly, patient-related risk factors (age, frailty, sarcopenia, nutritional deficiency, and metabolic comorbidities) frequently exert effects comparable to or greater than those of the medications themselves [25,33]. Clinical management must therefore incorporate individualized fracture risk estimation, lifestyle modification, fall-risk reduction, and correction of reversible contributors such as vitamin D deficiency, hypocalcemia, or chronic inflammatory states [33].

Given the multifactorial nature of osteoporosis, a proactive and individualized clinical approach is essential [25,33]. Comprehensive medication reconciliation should be performed for all patients presenting with low bone mass or fragility fractures, with particular attention to individuals exposed to long-term osteotoxic agents such as glucocorticoids, antiepileptic drugs, proton pump inhibitors, or selective serotonin reuptake inhibitors [33]. In patients with high-risk comorbidities—especially diabetes mellitus, chronic kidney disease, or hypertension, conditions known to impair bone material quality and accelerate calcium loss—baseline DXA screening may warrant earlier initiation than current guidelines recommend [33,162,168,169]. Longitudinal monitoring of bone mineral density and bone turnover markers can aid in assessing treatment response and identifying early skeletal deterioration [25,33]. Ultimately, a comprehensive approach that addresses both underlying comorbidities and medication-related skeletal toxicity is fundamental to long-term osteoporosis care.

Finally, it must be emphasized that many mechanistic insights summarized in this review originate from preclinical models, and large-scale human studies validating these pathways remain limited. Important uncertainties persist regarding dose–response relationships, exposure thresholds required to induce skeletal toxicity, and the reversibility of drug-induced molecular alterations [39]. The dynamic interplay between oxidative stress, immune activation, and disrupted osteoblast–osteoclast–osteocyte communication represents a major frontier for future investigation [50]. Addressing these gaps will be essential for refining risk stratification, improving therapeutic decision-making, and advancing precision medicine approaches in the management of osteoporosis [33,50].

## 8. Conclusions

Osteoporosis resulting from postmenopausal estrogen deficiency and diverse pharmacologic exposures—including glucocorticoids, bisphosphonates, denosumab withdrawal, selective serotonin reuptake inhibitors, proton pump inhibitors, heparin, and antiepileptic drugs—represents a heterogeneous clinical entity underpinned by convergent disruptions in skeletal homeostasis. Despite distinct molecular origins, these conditions commonly impair bone remodeling through dysregulation of the RANKL/OPG axis, suppression of Wnt/β-catenin signaling, reduced osteoblast differentiation, enhanced osteoclast activation, and increased oxidative stress. The cumulative effect is an imbalance between bone resorption and formation, ultimately compromising bone strength and increasing fracture susceptibility.

Understanding these mechanistic pathways carries significant therapeutic implications. Glucocorticoid-induced osteoporosis characteristically produces a low-turnover phenotype, often necessitating anabolic therapy, while SSRIs, PPIs, heparin, and antiepileptic drugs demand vigilant monitoring and individualized metabolic support. Denosumab withdrawal exemplifies a RANKL-driven osteolytic rebound requiring timely antiresorptive transition. Long-term bisphosphonate therapy highlights the risks of excessive remodeling suppression and microdamage accumulation, underscoring the need for periodic reassessment and potential drug holidays.

Moreover, comorbid conditions—particularly diabetes mellitus, hypertension, and other chronic systemic disorders—interact with these drug-induced pathways and further modulate skeletal vulnerability. Incorporating comorbidity profiles, medication exposures, and molecular mechanisms into clinical decision-making is therefore essential.

Future work should prioritize the identification of early biomarkers of skeletal toxicity, delineation of reversible versus irreversible drug effects, and evaluation of emerging interventions such as anti-sclerostin therapies, siRNA-based gene modulation, and digital adherence technologies. Through integration of mechanistic insight, comorbidity assessment, and precision medicine strategies, clinicians may better prevent fractures, preserve bone quality, and improve long-term outcomes in patients affected by both physiologic and medication-associated osteoporosis.

## Figures and Tables

**Table 1:** Comprehensive risk factors for osteoporosis and fragility fractures.

Category	Risk Factor
Non-modifiable	Advanced age
	Female sex
	Early menopause (<45 years)/menopause
	Genetic predisposition (ESR1, COL1A1, etc.)
	Family history of osteoporosis or fractures
Modifiable	Physical inactivity/sedentary behavior
	Low BMI (<18.5)
	Low calcium intake
	Vitamin D deficiency
	Smoking
	Excessive alcohol consumption
Comorbidities	Inflammatory diseases
	Inflammatory bowel disease
	Chronic kidney disease
	Celiac disease and other malabsorption disorders
	Chronic liver disease
	Dementia
	Chronic obstructive pulmonary disease (COPD)
	Systemic lupus erythematosus (SLE)
	Rheumatoid arthritis (RA)
	Multiple myeloma (MM)
	Monoclonal gammopathy of undetermined significance (MGUS)
	Thalassemia
	Mastocytosis
Medication-induced	Glucocorticoids
	Aromatase inhibitors
	Denosumab withdrawal
	Long-term bisphosphonates
	SSRIs
	Proton pump inhibitors
	Heparin
	Antiepileptic drugs (CYP450-inducing)
	Thiazolidinediones (PPAR-γ agonists)
	Warfarin
	Antiandrogens
	Chemotherapeutic agents
	Anticancer agents
Endocrine/metabolic factors	Hyperparathyroidism
	Hypercortisolism
	Hypogonadism
	Type 1 diabetes mellitus
	Type 2 diabetes mellitus
	Hyperthyroidism
	Acromegaly

**Table 2:** Summary of Key Molecular Mechanisms Driving Postmenopausal Osteoporosis (PMOP).

Key Mediators	Mechanism of Action	Impact on Bone Remodeling
Estrogen receptor signaling		
ERα ↓	Upstream trigger Estrogen deficiency reduces ERα activity, the central master switch of bone homeostasis. Transcriptional control Removes suppression on osteoclastogenic genes (e.g., RANKL) and reduces promotion of osteogenic genes.	Initiation of dysregulation Triggers the downstream cascades involving RANKL, Wnt, and inflammatory cytokines.
RANKL/OPG signaling		
RANKL ↑ OPG ↓	Ligand-receptor interaction Increased RANKL binds to RANK receptors on osteoclast precursors; decreased OPG reduces the decoy effect. Differentiation Directly stimulates osteoclast differentiation, activation, and survival.	Dominant resorption Shifts the remodeling balance heavily toward bone resorption (High turnover).
Wnt/β-catenin signaling		
Sclerostin ↑ DKK1 ↑ β-catenin ↓	Inhibition Upregulated inhibitors (Sclerostin, DKK1) bind LRP5/6 receptors. Downregulation Blocks β-catenin stabilization, reducing Runx2 and Osterix expression.	Impaired formation Suppresses osteoblast differentiation and matrix deposition, preventing repair.
Immune and Inflammatory		
T-cells ↑ Cytokines ↑ (TNF-α, IL-1β, IL-6, IL-7)	T-cell activation Estrogen deficiency increases IL-7, promoting T-cell expansion. Synergy Pro-inflammatory cytokines stimulate RANKL expression and synergize with RANKL signaling.	Accelerated turnover Creates a pro-inflammatory microenvironment that perpetuates bone loss.
Oxidative stress		
ROS ↑ NF-κB ↑	Signaling activation ROS activate NF-κB to directly increase RANKL expression. Cellular damage ROS induces osteoblast apoptosis and impairs differentiation.	Uncoupled remodeling Simultaneous stimulation of resorption and suppression of formation.
Genetic and Epigenetic factors		
SNPs Non-coding RNAs	Susceptibility Genetic variations (FSNPs) modulate BMD and fracture risk. Regulation Epigenetic regulators modulate osteoblast/osteoclast lineage commitment.	Risk modulation Determines individual susceptibility to bone loss and severity of disease.

↑ and ↓ indicate relative increases or decreases compared with physiological baseline.

**Table 3:** Summary of key molecular mechanisms driving drug-induced osteoporosis.

Molecular Pathway/Target	Mechanism of Action	Impact on Bone Remodeling
Glucocorticoids		
Wnt/β-catenin signaling	Upregulates DKK1 and Sclerostin, inhibiting Wnt signaling and osteoblast differentiation.	Bone formation ↓↓↓
RANKL/OPG axis	Increases RANKL and decreases OPG expression; prolongs osteoclast lifespan.	Bone resorption ↑
PPAR-γ pathway	Promotes MSC differentiation into adipocytes instead of osteoblasts.	Marrow adiposity ↑
Apoptosis pathways	Direct induction of apoptosis in osteoblasts and osteocytes via ROS and caspase activation.	Bone quality ↓
Proton pump inhibitors (PPIs)		
Gastric acidity	Increases gastric pH, reducing solubility and absorption of calcium salts.	Secondary hyperparathyroidism
PHOSPHO1 enzyme	Inhibits PHOSPHO1 phosphatase activity, which is essential for initiation of matrix mineralization.	Mineralization defect
Antiepileptics (AEDs)		
CYP450 induction	Induces hepatic CYP450 enzymes, accelerating catabolism of Vitamin D into inactive metabolites.	Hypocalcemia and PTH ↑
SSRIs		
Serotonin transporter (5-HTT)	Peripheral: Blocks 5-HTT in osteoblasts; extracellular 5-HT inhibits CREB/Runx2. Central: Increases sympathetic tone via hypothalamus.	Bone formation ↓ Bone resorption ↑
Thiazolidinediones (TZDs)		
PPARγ pathway (Primary Driver)	Activates PPARγ, suppressing Runx2 expression; diverts MSC differentiation towards adipocytes instead of osteoblasts.	Bone formation ↓ Marrow adiposity ↑
Wnt/β-catenin signaling	Upregulates sclerostin expression in osteocytes, inhibiting the canonical Wnt signaling pathway required for osteogenesis.	Bone formation ↓
RANKL/OPG axis	Increases RANKL expression and alters the RANKL/OPG ratio (secondary to PPARγ, enhancing osteoclastogenesis.	Bone resorption ↑
Opioids		
HPG axis (Primary Driver)	Inhibits GnRH release in the hypothalamus, leading to reduced LH/FSH and subsequent deficiency in sex hormones (Testosterone/Estrogen).	Bone resorption ↑
Opioid receptors (Direct)	Direct binding to receptors on osteoblasts inhibits proliferation and decreases Osteocalcin synthesis via cAMP pathway suppression.	Bone formation ↓
Aromatase inhibitors		
Estrogen depletion	Blocks conversion of androgens to estrogens, removing estrogenic inhibition on cytokines (IL-6, TNF-α) and RANKL.	High turnover bone loss
Heparin		
OPG sequestration	Electrostatically binds to OPG, preventing it from neutralizing RANKL.	Acute bone resorption
Denosumab withdrawal		
RANKL rebound	Cessation leads to a rapid surge in RANKL levels and fusion of osteomorphs (pre-osteoclasts).	Rapid bone resorption ↑↑
Osteoimmune signaling	Upregulation of osteoclast markers (TRAP, CTSK) due to loss of RANKL inhibition.	Vertebral fracture risk
Bisphosphonates(Long-term use)		
FPPS enzyme	Inhibits Farnesyl Pyrophosphate Synthase in osteoclasts, disrupting cytoskeleton and inducing apoptosis.	Bone resorption ↓↓
Bone turnover suppression	Severe suppression of remodeling leads to accumulation of microdamage (microcracks) and brittle bone matrix.	Atypical fractures

↑ and ↓ indicate relative increases or decreases compared with physiological baseline.

**Table 4:** Clinical risk factors, typical dosages, and treatment durations associated with drug-induced osteoporosis.

Drug Name	Patient Risk Factors (Comorbidities)	Dosage/Guideline Threshold	Clinical Relevance Duration
Glucocorticoids (GCs)	Underlying Inflammation (e.g., Rheumatoid arthritis, Systemic lupus erythematosus)	≥2.5 mg/day	>3 months Highest rate of bone loss in initial 3–6 months.
Proton pump inhibitors (PPIs)	Achlorydria Hypochlorhydria Malabsorption syndromes	Standard therapeutic dose	Continuous use for >1 year.
Antiepileptic drugs (AEDs)	Vitamin D Deficiency Institutionalized patients Polytherapy (Taking >2 AEDs)	Cumulative high dose (Dose-dependent risk)	Long term use
Antidepressants (SSRIs/TCAs)	Hyponatremia Concurrent Benzodiazepine use	Standard therapeutic dose (Dose-dependent risk)	Risk peaks within 1 month (TCAs) to 8 months (SSRIs). Risk diminishes toward baseline 1 year after cessation.
Thiazolidinediones (TZDs)	Postmenopausal Women Type 2 diabetes mellitus	Standard therapeutic dose	Increased fracture risk after 1 year treatment
Opioid	Hypogonadism	≥50–60 MME/day (Dose-dependent risk)	Risk peaks within 14 days due to CNS side effects leading to falls. Continuous use for >3 months.
Heparin	Pregnancy Primipara	>15,000–30,000 IU/day	>3 months (Usually reversible after cessation)
Denosumab (DEN)	Advanced CKD	Standard therapeutic dose	Increased risk of severe hypocalcemia in eGFR < 30 mL/min; requires calcium/vitamin D optimization and close monitoring. Subsequent antiresorptive therapy required upon discontinuation.
Bisphosphonates (BP)	Prior Chemotherapy	Standard therapeutic dose	First-line therapy for moderate fracture risk; may be used in high-risk patients when anabolic therapy is not feasible. After prolonged therapy (>5 years), reassessment and possible drug holiday may be considered in low-to-moderate risk patients.

## Data Availability

No new data were created or analyzed in this study. Data sharing is not applicable to this article.
